# Iptacopan for cold agglutinin disease: a case report with literature review

**DOI:** 10.3389/fimmu.2025.1672590

**Published:** 2025-10-03

**Authors:** Baozhi Fang, Hongbin Lu, Xiao Yu, Peng Wang, Yifei Zhou, Qiudan Shen, Muzhi Yuan, Mingen Lyu, Guangsheng He

**Affiliations:** ^1^ Department of Hematology, Affiliated Suzhou Hospital of Nanjing Medical University (Suzhou Municipal Hospital), Suzhou, China; ^2^ Wuxi Branch of Ruijin Hospital Shanghai Jiao Tong University School of Medicine, Wuxi, China; ^3^ Department of Hematology, The First Affiliated Hospital of Nanjing Medical University, Jiangsu Province Hospital, Nanjing, China

**Keywords:** autoimmune hemolytic anemia, cold agglutinin disease, iptacopan, lymphoplasmacytic lymphoma, rituximab

## Abstract

This study reports a case of cold agglutinin disease (CAD) secondary to lymphoplasmacytic lymphoma in a patient intolerant to rituximab plus bendamustine and with persistent uncontrolled hemolysis following zanubrutinib therapy. The addition of the complement C3 inhibitor iptacopan to a cyclophosphamide and dexamethasone regimen successfully controlled hemolysis and improved hemoglobin levels. Within one week of treatment, the patient achieved transfusion independence, with hemoglobin increasing from 67 g/L to 90 g/L by week 3 and 101 g/L by week 7, alongside normalized bilirubin levels and no adverse events. The follow-up period was 4 months, during which the patient showed sustained remission. These findings suggest that iptacopan can rapidly ameliorate hemolysis in CAD, warranting further investigation into its therapeutic potential.

## 1 Introduction

Autoimmune hemolytic anemia (AIHA) is a heterogeneous group of hemolytic anemias that are mediated by autoantibodies directed against one or more self-membrane antigens on erythrocytes. Based on the thermodynamic properties and optimal reactivity temperature of the causative autoantibodies, AIHA is classified into three distinct subtypes: warm AIHA, cold AIHA, and mixed-type AIHA. Among all AIHA cases, 15%–20% are cold AIHA, which can be further classified into cold agglutinin disease (CAD) and cold agglutinin syndrome (CAS). CAD is driven by monoclonal IgM binding to red blood cells, leading to activation of the complement pathway to produce C3b, which opsonizes red blood cells, inducing their destruction by phagocytes in the liver and causing extravascular hemolysis; downstream C5 may also be activated, thereby inducing membrane attack complex (MAC) formation and intravascular hemolysis (1). Glucocorticoids have poor efficacy in CAD. Although B cell-targeted therapies—including rituximab monotherapy or combination therapy with bendamustine or fludarabine—have improved efficacy, rituximab monotherapy is associated with high relapse rates. Combination treatment with bendamustine is accompanied by serious risks of agranulocytosis and infections, whereas combination treatment with fludarabine induces potent immunosuppression with a higher incidence of malignancy (2). In addition, there have been limited case reports of the use of bortezomib and Bruton’s tyrosine kinase inhibitors. Recently, complement inhibitors, including sutimlimab (which targets C1s) and pegcetacoplan (which targets C3), have demonstrated promising efficacy, but they have not yet been marketed in China. Iptacopan is a potent and selective oral complement factor B inhibitor that inhibits C3b production, thereby reducing C3b deposition on red blood cells and MAC formation, consequently controlling both intravascular and extravascular hemolysis (3). Herein, we present a case of CAD with lymphoplasmacytic lymphoma (MYD88 L265P mutation-negative) diagnosed by bone marrow biopsy that demonstrated a poor response to bendamustine in combination with rituximab and zanubrutinib. After switching to iptacopan combined with cyclophosphamide and dexamethasone, hemolysis was controlled and anemia improved.

To our knowledge, it was the first real-world evidence of iptacopan’s efficacy in CAD with concurrent lymphoma. This case demonstrates that selective C3b inhibition (via factor B blockade) can overcome resistance to anti-B cell therapies, which proposes a feasible regimen for regions lacking access to newer complement inhibitors.

## 2 Case presentation

**Figure 1:**
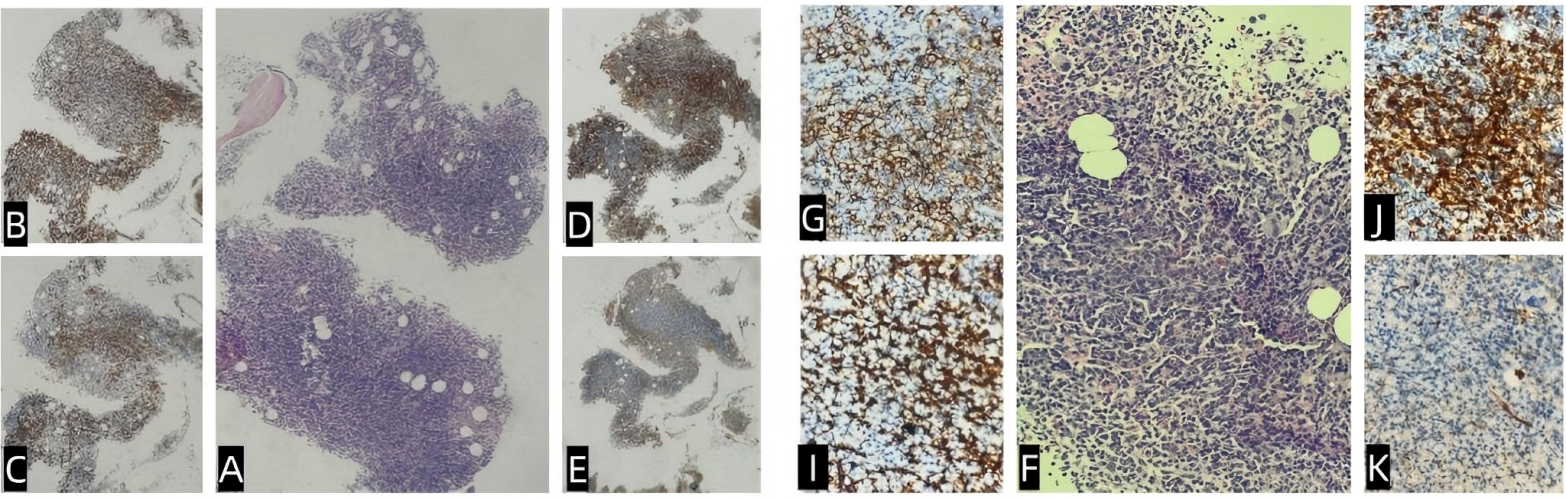
Bone marrow pathology and immunohistochemistry. The bone marrow exhibited active hematopoietic proliferation (hematopoietic capacity: approximately 90%), with diffuse or focal lymphoid hyperplasia and prominent plasma cells **(A, F)**. Granulocytic and erythroid cells showed low proliferation, whereas megakaryocytes were hyperplastic, accompanied by focal mild fibrosis. Immunohistochemistry showed regions that were CD3^+^ (diffuse) and CD20^+^ (small foci) **(B, G)**; CD138^+^ (scattered/focal) **(C, I)**; Igκ^+^ (focal) **(D, J)**; Igλ^+^ (focal) **(E, K)**; CD5^+^ (diffuse); CD10^+^ (stromal); and CD23^−^, LEF-1^−^, CD103^−^, cyclin D1.

A 44-year-old male presented with a one-year history of intermittent dizziness and generalized weakness. He also reported exertional dyspnea and occasional chest discomfort during physical activity. On physical examination, the following findings were noted. The patient’s pulse rate was 132 beats per minute. The patient exhibited a markedly anemic appearance, and jaundice was evident in both the skin and sclera. Palpation revealed splenomegaly, with the spleen edge palpable 3 cm below the left costal margin. No other distinct positive physical signs were identified. This patient had no prior history of hypertension, coronary heart disease, or urticaria. Before the onset of the illness, there was no record of previous infections or vaccinations. Both his personal and family medical histories were unremarkable. Additionally, there were no concomitant medications being taken. Laboratory tests revealed the following levels: white blood cells, 2.20 × 10^9^/L; absolute neutrophil count, 0.96 × 10^9^/L; hemoglobin, 45 g/L; platelet count, 48 × 10^9^/L; reticulocyte percentage, 3.7%; total bilirubin, 45 μmol/L; indirect bilirubin, 28 μmol/L; and lactate dehydrogenase, 372 U/L. Ferritin, folate, and vitamin B12 levels were within normal range. Flow cytometry analysis showed negative staining for fluorescent aerolysin (FLAER). Haptoglobin was <3.0 mg/dL, plasma-free hemoglobin was 28.5 mg/L, the direct antiglobulin test (Coombs) showed anti-C3d positivity (1:256), the cold agglutinin titer was 1:64, and serum IgM was 6.27 mg/L (**Table 1**). Immunofixation electrophoresis demonstrated monoclonal IgM-κ type M protein. Abdominal ultrasound revealed splenomegaly (163 mm × 61 mm), while whole-body CT showed no evidence of lymphadenopathy. Bone marrow morphology demonstrated lymphocytosis, and flow cytometry identified 1.8% CD5^-^CD10^-^ monoclonal B lymphocytes. Weakly clonal IgH gene rearrangement was detected along with clonal Igκ gene rearrangement. By bone marrow immunohistochemistry, we detected areas that were CD34^+^ (vascular), CD117^+^ (focal), CD3^+^ (diffuse), CD20^+^ (small foci), Pax-5^+^ (diffuse/focal), CD138^+^ (scattered/focal), Igκ^+^ (focal), Igλ^+^ (focal), CD5^+^ (diffuse), CD10^+^ (stromal), and CD23^−^, LEF-1^−^, CD103^−^, cyclin D1 (CCND1)^−^, and CD79a^+^ (all small foci). Bone marrow biopsy pathology revealed high levels of B lymphocytes with monoclonal plasma cell proliferation, suggestive of lymphoplasmacytic lymphoma (**Figure 1**). The patient was negative for both the *MYD88 L265P* and *CXCR4* gene mutations. There were no abnormalities in the chromosome karyotype. A final diagnosis of lymphoplasmacytic lymphoma associated with cold agglutinin disease (CAD) was established.

**Table 1:** Initial laboratory findings of the patient.

Parameter	Value	Reference range	Unit
Hematology			
White Blood Cells (WBC)	2.2	3.5–9.5	×10^9^/L
Absolute Neutrophil Count (ANC)	0.96	1.8–6.3	×10^9^/L
Hemoglobin (Hb)	45	130–175	g/L
Platelet Count	48	125–350	×10^9^/L
Reticulocyte Percentage	3.70%	0.5–1.5	%
Biochemistry			
Total Bilirubin	45	0-23	μmol/L
Indirect Bilirubin	28	0-23	μmol/L
Lactate Dehydrogenase (LDH)	372	120–250	U/L
Haptoglobin	<3.0	32–205	mg/dL
Plasma-Free Hemoglobin	28.5	0-40	mg/L
Immunology			
Direct Antiglobulin Test (Anti-C3d)	1:256	Negative	–
Cold agglutinin titer	1:64	Negative	–
Serum IgM	6.27	0.4–2.3	mg/L

**Figure 2:**
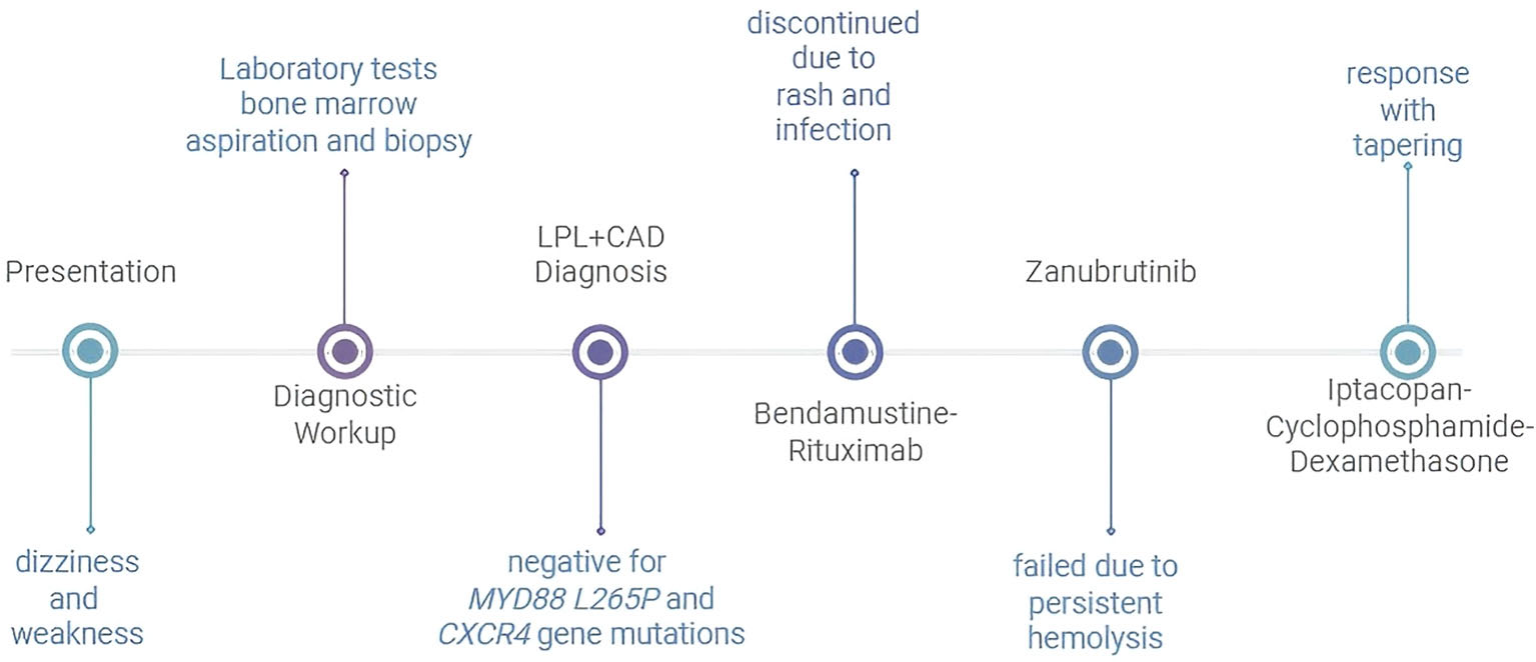
Timeline of diagnostic events and treatments LPL, lymphoplasmacytic lymphoma; CAD, cold agglutinin disease.

**Figure 3:**
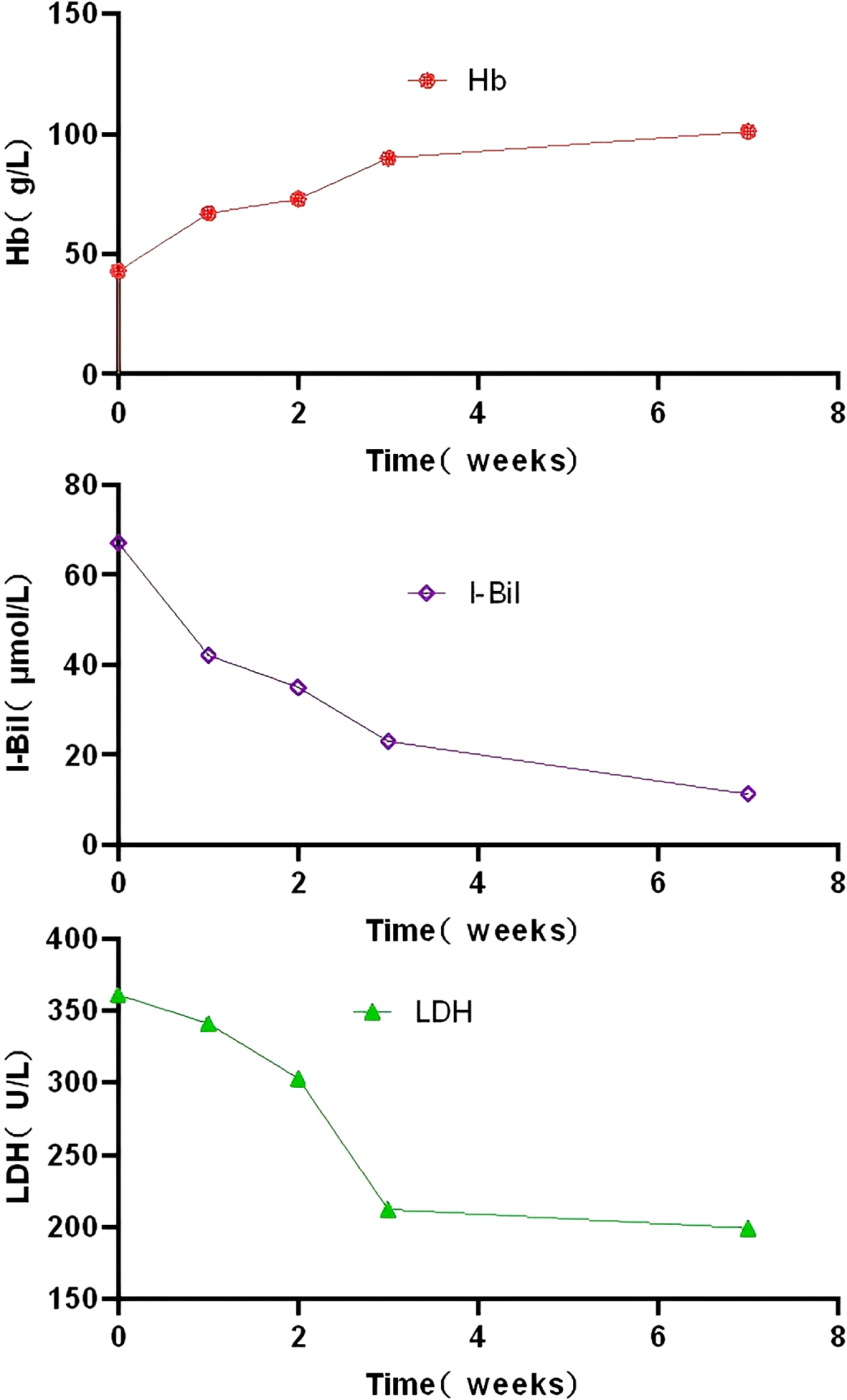
Changes in hemoglobin, indirect bilirubin levels and lactate dehydrogenase following iptacopan treatment. Hb, hemoglobin; I-Bil, indirect bilirubin; LDH,lactate dehydrogenase.

The patient received bendamustine 90 mg/m² on days 1–2 and rituximab 375 mg/m² on day 1. Extensive blistering and desquamative rashes with secondary infection developed on both feet and pretibial areas following chemotherapy. The bendamustine–rituximab regimen was discontinued and replaced by zanubrutinib 160 mg twice daily. Despite treatment, laboratory testing revealed persistent hemolysis: hemoglobin was 43 g/L, reticulocyte percentage was 3.8%, total bilirubin was 74 μmol/L, indirect bilirubin was 67 μmol/L, and lactate dehydrogenase was 361 U/L. Haptoglobin was <3.0 mg/dL and plasma-free hemoglobin was 8.9 mg/L. The direct antiglobulin test remained positive for anti-C3d (1:256). The patient remained transfusion-dependent, requiring two units of red blood cells per week. Zanubrutinib was discontinued. Treatment was initiated with iptacopan 200 mg twice daily, cyclophosphamide 100 mg once daily, and dexamethasone 6 mg once daily. After 1 week of treatment, the patient achieved transfusion independence with a hemoglobin level of 67 g/L. Hemoglobin rose to 90 g/L at 3 weeks and the regimen was adjusted to iptacopan 200 mg once daily, cyclophosphamide 50 mg once daily, and dexamethasone 3 mg once daily. The timeline of the diagnostic events and the treatments used during the whole period was as follows (**Figure 2**). Seven weeks later, hemoglobin was 101 g/L (**Figure 3**), bilirubin was normal, and the anti-C3d titer was 1:16. The regimen was further tapered to iptacopan (200 mg every other day) and cyclophosphamide (50 mg every other day), while dexamethasone was discontinued. The follow-up period was 4 months, during which the patient showed sustained remission. No drug-related adverse events were observed.

## 3 Discussion

It was previously believed that cold agglutinin disease (CAD) occurred as a primary condition without an underlying disorder. However, it is now well established that approximately 90% of CAD cases are associated with a clinically significant monoclonal gammopathy. Recent studies have described a distinct entity termed CAD-related lymphoproliferative disease, characterized by micronodular lymphocytic bone marrow infiltrates involving less than 10% of marrow cellularity in patients with CAD (2). The MYD88 L265P mutation, which is found in nearly all cases of lymphoplasmacytic lymphoma (LPL), is notably absent in CAD (1). In the present case, the patient exhibited cold agglutinin-positive autoimmune hemolytic anemia (AIHA) with a monoclonal IgM-κ paraprotein and a bone marrow biopsy suggestive of lymphoplasmacytic lymphoma. The absence of the *MYD88 L265P* mutation is consistent with CAD secondary to lymphoplasmacytic lymphoma.

**Figure 4:**
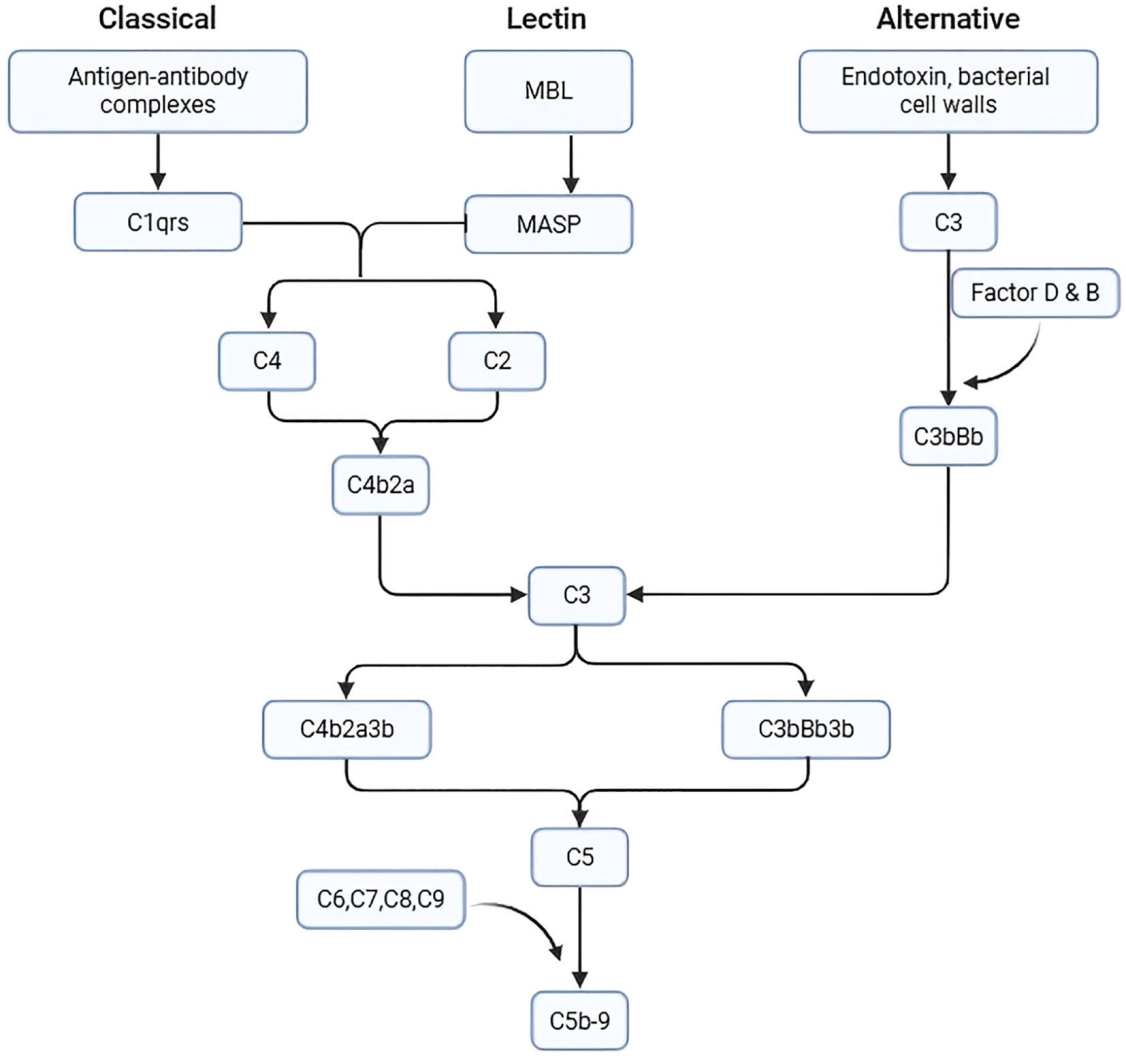
Complement cascade MBL, Mannose-binding lectin; MASP, MBL-associated serine protease.

CAD is an AIHA driven by cold IgM antibodies; it is characterized by complement C3b-mediated hemolysis. IgM binds to erythrocytes at lower temperatures—typically in peripheral circulation, such as the extremities—triggering recruitment of the C1 complex and activation of the classical complement pathway (4). Once C1 is activated, it cleaves C4 and C2 to form C3 convertase (C4b2a), which subsequently cleaves C3 into C3a (a soluble anaphylatoxin) and C3b (which binds to cell membranes). This biphasic pattern of complement activation has also been observed in human serum sickness nephritis: the early phase (within 72 hours) is predominantly classical pathway–driven, while the late phase (>7 days) involves significant amplification through the alternative pathway (5). After activation of the classical pathway, the alternative pathway can be amplified to produce more C3b (**Figure 4**). Erythrocytes opsonized with C3b are cleared by the reticuloendothelial system, primarily in the liver, via phagocytosis by monocytes, macrophages, and neutrophils, resulting in extravascular hemolysis. C3b can also act as a C5 convertase, cleaving C5, which further binds to C6, C7, C8, and C9 to form a MAC, resulting in intravascular hemolysis.

Aggressive treatment is required for patients with CAD with severe hemolysis and persistent hemoglobin <100 g/L, and for patients with CAD with severe microcirculatory symptoms. Glucocorticoids have limited efficacy in CAD, with only 15%–20% of patients achieving an initial response. Since C3b-mediated erythrocyte destruction primarily occurs in the liver, splenectomy is ineffective and not recommended. Instead, treatment strategies focus on targeting B lymphocytes or plasma cells responsible for autoantibody production. The overall response rate (ORR) of patients with CAD treated with rituximab is approximately 50%, but most responders relapse (6, 7). The combination of rituximab and bendamustine increases the ORR to 75% but increases the risk of neutropenia and serious infections (8). Treatment with rituximab plus fludarabine resulted in a 38% complete response (CR) rate, with a median duration of response of 77 months; however, 31% of those achieving CR developed secondary malignancies during follow-up (7). Given these limitations, there is a clear need for continued development of novel therapies in CAD to improve efficacy while minimizing long-term toxicity. In a small retrospective study, 13 patients with CAD/CAS were treated with ibrutinib, with an ORR of 100%, including 12 patients with CR (9). In a Phase 2 prospective study, bortezomib was evaluated in relapsed CAD and achieved a CR rate of 15.8% and an ORR of 31.6% (10).

Because CAD hemolysis is mediated by C3b, complement inhibitors have become another treatment option for CAD. An open-label phase 2 study shows that eculizumab significantly reduced hemolysis and transfusion requirement in patients with CAD (11). Two recent prospective trials demonstrated that sutimlimab, a C1s inhibitor targeting the classical complement pathway, is highly effective and safe in CAD/CAS. It induced rapid responses in 54%–73% of patients, with a mean hemoglobin increase of 26 g/L and significant improvement in quality of life, particularly in Functional Assessment of Chronic Illness Therapy–Fatigue scores. The therapeutic effect was sustained with continued treatment, but hemolytic anemia could relapse within weeks of discontinuation (12, 13). Gavriilaki et al. reported an elderly patient with warm autoimmune hemolytic anemia (wAIHA) refractory to three prior therapies. Pegcetacoplan treatment led to a significant hemoglobin (Hb) increase from 53 g/L to 107 g/L (14). In an open-label phase 2 study of the C3 inhibitor pegcetacoplan in CAD and mAIHA, patients were randomized to receive either 270 mg/day or 360 mg/day of pegcetacoplan for up to 48 weeks. Among 30 patients screened (24 receiving ≥1 dose), 10 completed 270- and 360-mg daily dosing in CAD (5 patients each), whereas 4 (270 mg) and 3 (360 mg) participants completed dosing in wAIHA. The median (interquartile range) change from baseline Hb for the CAD and wAIHA total groups was 2.4 and 1.7 g/dL, respectively (15) (**Table 2**). Complement inhibition has increasingly become the standard of care for CAD.

**Table 2:** Clinical trial data of complement inhibitors in cold agglutinin disease.

Authors	Year	Complement inhibitor	Target	Study type	Number	Treatment dose	Clinical response
Röth A,et al (11)	2018	Eculizumab	C5 inhibitor	phase 2 trial	13	600 mg/week ×4, then 900 mg q2w	LDH (572 to 334 U/L); Hb (9.35 to 10.15 g/dL)
Röth A,et al (12)	2021	Sutimlimab	C1s inhibitor	phase 3 trial	24	6.5 g (BW <75 kg) 7.5 g (BW ≥75 kg)	Hb>12g/dL or ΔHb>2.0g/dL:54%
Röth A,et al (13)	2022	Sutimlimab	C1s inhibitor	phase 3 trial	42	6.5 g (BW <75 kg) 7.5 g (BW ≥75 kg)	ΔHb>2.0g/dL: 72.7% in sutimlimab group vs. 10% in placebo group
Roman E,et al (14)	2025	Pegcetacoplan	C3 inhibitor	phase 2 trial	10	270 mg/day 360 mg/day	ΔHb: 2.4g/dL

BW, bodyweight; Hb, hemoglobin; LDH, lactate dehydrogenase; ΔHb, post-treatment hemoglobin level - baseline hemoglobin level.

Iptacopan is a potent and selective oral inhibitor of complement factor B. It reduces C3 cleavage into C3b, controls the amplification of classically generated C3b via the alternative pathway, and decreases overall C3b production, thereby suppressing both extravascular hemolysis (mediated by C3b-opsonized erythrocyte destruction in the liver) and intravascular hemolysis (caused by MAC formation). Iptacopan has shown promise in several complement-mediated disorders. Iptacopan can effectively control extravascular hemolysis and residual intravascular hemolysis secondary to C5 inhibitor therapy in patients with paroxysmal nocturnal hemoglobinuria (3). In the Phase III APPLY-PNH trial, by the 48-week follow-up, 51 out of 59 patients (86%) experienced an elevation in hemoglobin concentration of at least 2 g/dL compared to the baseline. Furthermore, 40 out of 59 patients (68%) achieved a hemoglobin concentration of at least 12 g/dL. Similarly, in the APPOINT-PNH study at the 48 - week time point, a total of 38 out of 39 patients (97%) showed an increase in hemoglobin concentration of at least 2 g/dL from the baseline. Additionally, 31 out of 39 patients (79%) reached a hemoglobin concentration of at least 12 g/dL (16). The Phase III APPLAUSE-IgAN trial revealed an impressive outcome: patients receiving iptacopan had a striking 38.3% decline in proteinuria levels (17). Meanwhile, iptacopan is under rigorous investigation in Phase III trials targeting other complement - mediated pathologies, namely C3 Glomerulopathy (C3G) and Atypical Hemolytic Uremic Syndrome (aHUS) (18). Findings from clinical trials suggest that iptacopan is, overall, well - endured by patients.

Considering that complement is also involved in the hemolytic mechanism of cold agglutinin disease (CAD), our patient with CAD had features of both extravascular and intravascular hemolysis, was intolerant to bendamustine–rituximab therapy, and experienced worsening of hemolysis during treatment with zanubrutinib. At present, sutimlimab and pegcetacoplan are not available in China, so we used iptacopan to control C3b production. Two weeks prior to initiating iptacopan treatment, the patient was administered vaccines against *Neisseria meningitidis* and *Streptococcus pneumoniae*. Additionally, since CAD is driven by lymphoplasmacytic lymphoma, we administered dexamethasone combined with cyclophosphamide to target the underlying disease. The patient exhibited a rapid clinical response, becoming transfusion-independent within one week, with hemoglobin rising to 90 g/L at 3 weeks and 101 g/L at 7 weeks. His bilirubin levels decreased to normal. The patient has maintained treatment for 3 months with good tolerance and no adverse effects. After acute hemolysis was controlled, the patient was initiated on the bendamustine-rituximab regimen for maintenance therapy due to financial considerations. These findings suggest that exploring therapies that combine complement inhibitors with lymphoma-targeted regimens represents a promising direction for the treatment of CAD secondary to lymphoproliferative disorders.

Our study has several limitations. First, as a single-case report, our findings may lack generalizability to broader patient populations. Second, the follow-up period was relatively short (4 months), precluding a comprehensive assessment of long-term outcomes.

## Data Availability

The original contributions presented in the study are included in the article/supplementary material, further inquiries can be directed to the corresponding author/s.
